# ICD-11 complex posttraumatic stress disorder and subclasses of borderline personality disorder in a South Korean adult population with childhood abuse experiences: a latent class analysis

**DOI:** 10.1186/s40479-023-00242-z

**Published:** 2023-12-15

**Authors:** Jisu Lee, Hyunjung Choi

**Affiliations:** Chungbuk National University, Cheongju, South Korea

**Keywords:** Complex posttraumatic stress disorder, Borderline personality disorder, Latent class analysis, Pathological personality traits, Childhood abuse

## Abstract

**Background:**

Complex posttraumatic stress disorder (CPTSD) and borderline personality disorder (BPD), which are distinctive diagnoses, share the common risk factor of childhood abuse experiences. However, additional evidence is needed to determine which factors contribute to the manifestation of different symptoms.

**Method:**

Participants were 499 South Korean early and midlife adults with primarily college level education who reported experiences of childhood abuse. They were enrolled from an online panel using a stratified sampling considering gender, age, and residence information. A latent class analysis (LCA) was conducted to identify the patterns of CPTSD and BPD symptoms. We adopted a three-step LCA to compare types of childhood abuse, invalidating environments, attachment styles, and pathological personality traits among different classes.

**Results:**

The LCA revealed four classes. Class 1 showed the highest scores in all symptoms and risk factors. Class 2 was distinguished from Class 3 by the externalizing versus internalizing associated pathological personality traits. Class 3 experienced high emotional neglect in addition to other types of abuse and it also showed an additional avoidant attachment style. Class 4 showed low symptomatology.

**Conclusion:**

Class 1 was named as a CPTSD and BPD “comorbid” class, Class 2 as an “externalizing BPD” class, Class 3 as an “avoidant BPD” class, and Class 4 as a “low symptom” class. Childhood abuse may heighten the risk for high comorbidity of CPTSD and BPD as well as externalizing-internalizing subgroups of BPD. Beyond the identification of CPTSD and BPD, assessing attachment styles and pathological personality traits based on dimensional approaches would benefit the tailoring of effective treatment.

## Background

Childhood abuse and neglect have been shown to serve as risk factors for a range of psychopathologies [1], including complex posttraumatic stress disorder (CPTSD) [2, 3] and borderline personality disorder (BPD) [4–6]. *The International Classification of Diseases* (ICD-11) CPTSD consists of posttraumatic stress disorder (PTSD) symptoms such as re-experiencing, avoidance, and heightened sense of threat, along with disturbances in self-organization (DSO) symptoms, which are characterized by affect dysregulation, negative self-concept, and disturbed relationships [7]. Meanwhile, BPD is characterized by pervasive dysregulation in relationships, emotions, cognition, and identity [8].

While the symptoms of BPD and DSO overlap with each other, recent discussions have agreed that CPTSD and BPD can be differentiated. Using an exploratory factor analysis, the ICD-11 PTSD, DSO, and BPD symptoms were shown to have distinct constructs [9, 10]. In a network analysis, CPTSD and BPD were clearly separated, as they only overlapped in terms of the affect dysregulation symptom [11]. In the results of a latent class analysis (LCA) conducted among women with childhood abuse histories, despite the presence of overlapping symptoms, BPD was distinguished from CPTSD [3]. CPTSD, PTSD, and BPD were all distinguished from each other in a latent profile analysis (LPA) among community sample women with at least one potential lifetime trauma experience [12]. Studies have shown that, whereas DSO is related to emotional and interpersonal avoidance, BPD is more characterized by unstable interpersonal relationships and sense of self, impulsivity, and reactive emotional responses [3, 9]. Consistently, dissociative symptoms in CPTSD have referred to relatively prolonged emotional numbing, which are at higher levels than in BPD, while they are responses to temporary stress in BPD [12].

Some studies have shown that CPTSD and BPD can be highly comorbid. For example, in an LCA with a general population sample with sexual trauma, a distinctive CPTSD and a PTSD class was distinguished in addition to classes of those comorbid with BPD [13]. Other studies have found comorbid classes of CPTSD with varying symptom severity in BPD, where a distinguished class has not been found, both in a multiple traumatized treatment-seeking people [14] and a young people sample with lifetime trauma [15].

The results showed that CPTSD and BPD were distinctive, while the phenomena of the symptoms differed even for the similarities of the symptoms [3, 16]. However, no population characteristics of type of trauma emerged as distinctive risk factors for CPTSD and BPD. For instance, childhood interpersonal violence is a common risk factor for both CPTSD and BPD [3, 9], and most populations are multiply traumatized, because trauma experience itself may be a risk factor for further traumatization later in life [e.g., 13–15]. It is also necessary to understand the differential trajectories of how CPTSD and BPD develop. Therefore, in the present work, we focused on attachment styles and pathological personality traits based on the dimensional perspective of psychopathology [e.g., 17, 18] in people who have experienced childhood abuse.

Childhood abuse is a risk factor for insecure attachment, including both anxious and avoidant attachment. Attachment style has also been shown to mediate interpersonal problems and emotional maladjustment in later life [19–22]. Anxious attachment is characterized by both a strong desire to be close and concerns about being rejected in relationships. Meanwhile, avoidant attachment is associated with fear and avoidance of getting close to or depending on others [19]. The fear of abandonment that is commonly seen in BPD closely resembles the pattern of anxious attachment. A meta-analysis showed that BPD is more strongly associated with anxious than avoidant attachment [23]. The attachment style in CPTSD symptoms appear to be more complicated. In one study, childhood trauma related CPTSD symptoms have been found to be associated with both anxious and avoidant attachment [24], while multiple studies have repeatedly confirmed the strong relationship between CPTSD and anxious attachment [10, 25–27].

Differences in the personality traits of CPTSD and BPD can be identified based on the Hierarchical Taxonomy of Psychopathology (HiTOP) model developed by Kotov and colleagues [17, 18]. The HiTOP directs a paradigm shift from a categorical model to the dimensional model of psychopathology and aim to identify a transdiagnostic variable to be targeted in treatment. This model explains the dimensional features related to PTSD and BPD according to the *Diagnostic and Statistical Manual of Mental Disorders* (DSM) [8]. PTSD was explained in terms of the internalizing spectrum, while BPD was classified along both the internalizing and antagonistic externalizing dimensions [18]. HiTOP contains spectra that correspond to the five domains of the pathological personality traits presented in the Alternative Model for Personality Disorders (AMPD) in DSM-5 [8]. The AMPD concept has previously been used to understand not only personality disorders but also other mental disorders [28, 29]. In Møller et al., DSO symptoms showed strong correlations with the maladaptive personality trait domains of negative affectivity, detachment, and psychoticism [29]. Gamache and colleagues conducted an LPA using AMPD concepts as indicators and identified sub-profiles of BPD along a dimension of severity and different categories of personality traits [30].

The current study aimed to investigate how the symptoms of CPTSD and BPD manifest in a sample of Korean adults with childhood abuse experiences. The attachment styles and pathological personality traits associated with each latent syndrome were compared. We also compared being raised in an invalidating environment which has been discussed as a risk factor for BPD involving neglect and mislabeling of a child’s emotions, thoughts, and behaviors; reinforcement of maladaptive emotional expression; and a lack of opportunities to develop problem-solving strategies [31]. The research hypotheses are as follows: First, the CPTSD and BPD classes are expected to be distinguishable using LCA. Second, both CPTSD and BPD are expected to be highly related to child abuse and invalidating environments. Third, the CPTSD and BPD classes are each expected to show both high avoidant and anxious attachment, while the BPD class is expected to show less avoidant attachment. Finally, the two classes are expected to differ in terms of pathological personality traits. CPTSD is expected to show high scores in the negative affectivity domain corresponding to the internalizing spectra as well as the detachment domain in relation to chronic interpersonal avoidance. In particular, BPD is expected to be associated with the disinhibition domain.

## Methods

### Participants and procedures

The present study was approved by the Institutional Review Board of the authors’ institution. Data were collected from an online panel of adults who reported having at least one childhood abuse experience. Stratified sampling was conducted while considering gender, age, and residence information [32] based on South Korean population data from Statistics Korea. The exclusion criteria were individuals who were under the age of 19, those who did not use Korean as their first language, and those with no experiences of childhood abuse. We excluded data based on a logical criterion that informs the validity of the sample, as suggested by Kramer and colleagues [33]. We considered data to be invalid when responding to extreme values in opposite directions to items with identical content regarding childhood abuse and an invalidating environment. Out of 14,220 people, 13,511 were excluded due to the absence of a history of childhood abuse, and invalid data from 172 individuals were excluded. A second data collection was conducted adding 171 participants. Eventually, data of a total of 499 participants were analyzed.

There were 256 men (51.3%) and 243 women (48.7%) in the sample. In terms of age, participants were in their 20s through 50s, with the proportions being 22.2% in their 20s, 22% in their 30s, 27.5% in their 40s, and 28.3% in their 50s. In terms of education, 107 people were high school graduates or lower (21.4%) whereas 392 had college degrees and above (78.5%). The percentage of those with a college education was higher in our sample than that in the Korean general population (52%) [34], showing that our sample primarily represented college educated early and midlife adults with childhood abuse experiences. Regarding employment conditions, there were 330 regular workers (66.1%), 47 nonregular workers (9.4%), 34 self-employed workers (6.8%), 66 unemployed individuals (13.2%), and 22 students (4.4%).

Using the cutoff criteria [35], 31.7% of the sample reported physical abuse (PA), 28.3% reported emotional abuse (EA), 29.7% reported sexual abuse (SA), 28.5% reported physical neglect (PN), and 37.5% reported emotional neglect (EN).

### Measures

#### International trauma questionnaire, ITQ

The International Trauma Questionnaire (ITQ) measures PTSD and DSO symptoms according to ICD-11 using a 5-point Likert scale. The reliability coefficient Cronbach’s α was above 0.77 for all PTSD and DSO symptoms, except for the avoidance items [36]. Cronbach’s α values for the Korean version were 0.92 for PTSD symptoms and 0.91 for DSO symptoms [37]; in the present study, they were 0.95 and 0.94, respectively.

#### Borderline symptom list-short form, BSL-23

The Borderline Symptom List-short form (BSL-23) measures symptoms of BPD based on DSM-IV rated on a 5-point Likert scale [38]. The items capture symptoms such as self-perception, affect regulation, self-destruction, dysphoria, loneliness, intrusions, and hostility. We used the Korean version validated by Kang et al. [39]. Cronbach’s α was 0.97 in the development study [40] and 0.98 in both Kang et al. [39] and the current study.

#### Child trauma questionnaire-short form, CTQ-SF

The Child Trauma Questionnaire-Short Form measures the frequency of five types of child abuse, including PA, EA, SA, PN, and EN, on a 4-point Likert scale [41]. The Cronbach’s α values of the Korean version were 0.88 for PA, 0.82 for EA, 0.87 for SA, 0.68 for PN, and 0.86 for EN [42]. In the current study, the Cronbach’s α values were 0.85 for PA, 0.84 for EA, 0.93 for SA, 0.67 for PN, and 0.92 for EN.

#### Invalidating childhood environment scale, ICES

The Invalidating Childhood Environment Scale (ICES) assesses parental invalidation and invalidating family types on a 5-point Likert scale [43]. We used the parental invalidation scales from the Korean version [44]. The paternal and maternal Cronbach’s α values were respectively 0.80 and 0.77 in the original version, 0.87 and 0.86 in Korean version, and 0.75 and 0.82 in the current study.

#### Experiences in close relationships-revised, ECR-R

The Experiences in Close Relationships-Revised (ECR-R) measures adult attachment styles [45]. The current study used the Korean version validated by Kim [46]. The ECR-R consists of 18 items for anxious attachment and 18 items for avoidant attachment, which are responded to on a 5-point Likert scale. In the Korean version, the Cronbach’s α values were 0.89 for anxious attachment and 0.85 for avoidant attachment; in the current study, they were 0.93 and 0.91, respectively.

#### Personality inventory for DSM-5, PID-5

The Personality Inventory for DSM-5 (PID-5) measures the pathological personality traits presented in the DSM-5 AMPD [47]. We used the Korean version validated by Shin and Hwang [48], which consists of 25 facets organized into five domains (negative affectivity, detachment, antagonism, disinhibition, and psychoticism). In total, 220 items are rated on a 4-point Likert scale (0: not at all ~ 3: very much). The reliability coefficients for each domain were as follows: Negative Affectivity 0.93, Detachment 0.96, Antagonism 0.95, Disinhibition 0.84, Psychoticism 0.96 [47]. In the current study, the coefficients were 0.97, 0.93, 0.96, 0.94, and 0.96, respectively.

### Analyses

An LCA, which is a structural equation modeling method that identifies unobserved structures in multivariate categorical data [49], was conducted using Mplus 8.0. Each of the three symptoms of PTSD (reexperience, avoidance, and heightened sense of threat) and DSO (affect dysregulation, negative self-concept, and disturbed relationship) were scored as present and non-present according to the algorithm suggested by Cloitre and colleagues [36], where any of the two items from each symptom is rated 2 or higher, meaning that the symptom is present. The latent variable of BPD symptoms was composed of each item of the BSL-23, where symptoms were defined as present when the score was 2 or higher.

To identify the optimal model, we compared the Akaike’s Information Criteria (AIC) [50], the Bayesian Information Criteria (BIC) [51], the Sample-Size Adjusted BIC (SSA-BIC) [52], and entropy values. When the AIC, BIC, and SSA-BIC values are smaller [53] and the entropy value is 0.8 or higher, the classification accuracy is considered to be good [54]. If the Lo-Mendell-Rubin Adjusted likelihood ratio test (LMR-A) and the Bootstrap Likelihood Ratio Test (BLRT) values are significant, then the k-level model is considered to be a better fit than the k-1 level model [55].

We used a three-step LCA to examine differences in the type of childhood abuse, invalidating environments, attachment style, and pathological personality traits across latent classes. A three-step method improves statistical power by using auxiliary observed variables to adjust classification errors in the parameter estimation process [56].

## Results

### Latent class analysis

The fit scores of each latent class model are presented in Table 1. We excluded the five-class model because the maximum likelihood was not repeatedly extracted in that model. The four-class model was selected, because it had the lowest values for BIC, SSA-BIC, and AIC, as well as significant relative fit indices.

**Table 1:** Latent class models and fit indices

Model	Log-likelihood	BIC	ssa-BIC	AIC	Entropy	LMR-A *p*-value	BLRT *p*-value
1 class	-9851.951	19884.068	19792.020	19761.902			
2 class	-6711.054	13788.652	13601.383	13540.109	0.979	0.000	0.000
3 class	-6184.318	12921.558	12639.067	12546.636	0.958	0.000	0.000
**4 class**	**-5951.737**	**12642.773**	**12265.061**	**12141.473**	**0.976**	**0.003**	**0.000**
5 class	-5768.696	12463.071	11990.137	11835.393	0.963	0.013	0.000

Note. BIC = Bayesian Information Criterion; ssa-BIC = sample-size adjusted BIC; AIC = Akaike Information Criterion; LMR-A = Lo–Mendell–Rubin Adjusted likelihood ratio test; BLRT = Bootstrap Likelihood Ratio Test

*5 class was excluded because the best log-likelihood value was not replicated

**Fig. 1:**
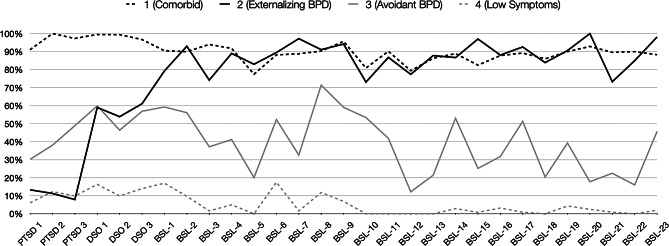
Symptom endorsement of complex posttraumatic stress disorder and borderline personality disorder items

Each class of symptom patterns is presented in Fig. 1, and the conditional response probabilities are presented in Table 2. If the response probabilities are above 70% or below 30%, the consistency within that class was considered to be high [57].

**Table 2:** Conditional response probabilities in each class

Symptoms		Class 1 (Comorbid) (*n* = 189)	Class 2 (Externalizing BPD) (*n* = 53)	Class 3 (Avoidant BPD) (*n* = 137)	Class 4 (Low Symptoms) (*n* = 120)
PTSD 1	Re-experiencing	0.91	0.13	0.30	0.06
PTSD 2	Avoidance	1.00	0.11	0.38	0.12
PTSD 3	Sense of threat	0.97	0.08	0.49	0.10
DSO 1	Affect dysregulation	1.00	0.59	0.60	0.16
DSO 2	Negative self-concept	0.99	0.54	0.46	0.10
DSO 3	Disturbed relationship	0.97	0.61	0.57	0.14
BSL-1	Difficulty in concentration	0.91	0.79	0.59	0.17
BSL-2	Helplessness	0.90	0.93	0.56	0.10
BSL-3	Losing mind and memory	0.94	0.74	0.37	0.02
BSL-4	Disgust	0.92	0.89	0.41	0.05
BSL-5	Thoughts of hurting oneself	0.78	0.83	0.20	0.00
BSL-6	Not trusting others	0.88	0.90	0.52	0.18
BSL-7	No right to live	0.89	0.97	0.33	0.02
BSL-8	Lonely	0.90	0.91	0.71	0.12
BSL-9	Pressured by inner tension	0.96	0.94	0.59	0.07
BSL-10	Scary images	0.81	0.73	0.53	0.00
BSL-11	Hated oneself	0.90	0.87	0.42	0.00
BSL-12	Wants to punish oneself	0.79	0.77	0.12	0.00
BSL-13	Distressful shame	0.86	0.88	0.21	0.00
BSL-14	Mood fluctuation	0.89	0.87	0.53	0.03
BSL-15	Distressed by voices or noises	0.83	0.97	0.25	0.01
BSL-16	Horrible effects being judged	0.88	0.88	0.32	0.03
BSL-17	Felt vulnerable	0.89	0.93	0.51	0.01
BSL-18	Fascinated with death	0.86	0.84	0.20	0.00
BSL-19	Felt meaningless	0.90	0.91	0.39	0.04
BSL-20	Fear of losing control	0.93	1.00	0.18	0.03
BSL-21	Disgusted with oneself	0.90	0.73	0.23	0.01
BSL-22	Felt distancing from oneself	0.90	0.85	0.16	0.00
BSL-23	Felt worthless	0.88	0.98	0.46	0.02
Mean of probabilities		0.90	0.76	0.40	0.05

Note. BSL-23 = Borderline Symptom List-short form

*The *p*-values of all post-hoc tests were less than 0.001 (*df* = 3)

To facilitate the identification of each class, the probable PTSD, CPTSD [36] and BPD [39] diagnoses among each class were calculated based on the diagnostic algorithm and cutoff points, and these are presented in Table 3. The scores of PTSD, DSO, and BPD symptoms were described together.

**Table 3:** Mean score of the ITQ and BSL-23 and probable diagnoses of CPTSD and BPD among classes

		Class 1 (Comorbid) (*n* = 189)	Class 2 (Externalizing BPD) (*n* = 53)	Class 3 (Avoidant BPD) (*n* = 137)	Class 4 (Low Symptoms) (*n* = 120)
*M* (*SD*)	ITQ (PTSD)	2.42 (0.61)	0.71 (0.45)	1.02 (0.74)	0.33 (0.38)
	ITQ (DSO)	2.57 (0.55)	1.34 (0.77)	1.46 (0.77)	0.43 (0.41)
	BSL-23	2.50 (0.42)	2.43 (0.45)	1.30 (0.39)	0.36 (0.25)
*n* (%)	PTSD	8 (4.2)	0 (0.0)	15 (10.9)	0 (0.0)
	CPTSD	153 (81.0)	0 (0.0)	2 (1.5)	0 (0.0)
	BPD	189 (100.0)	53 (100.0)	133 (97.1)	19 (15.8)

Note. ITQ = International Trauma Questionnaire; BSL-23 = Borderline Symptom List-short form

Class 1 showed high probabilities for symptoms of PTSD, DSO, and BPD. The majority of the people (n = 153, 81%) in Class 1 satisfied a probable diagnosis for CPTSD and 100% of those in Class 1 satisfied a probable diagnosis for BPD. Class 2 showed very low probabilities for PTSD symptoms and a high probability of 70% or more for all BPD symptoms. All people in Class 2 showed a probable diagnosis of BPD, but no CPTSD or PTSD. Class 3 showed moderate probabilities for PTSD symptoms and a high probability for the BPD symptom of loneliness. People in Class 3 showed low probabilities for other BPD symptoms, such as thoughts of hurting oneself, wanting to punish oneself, fascination with death, distressful shame, self-disgust, distressful voices and noises, fear of losing control, and distancing oneself. In Class 3, the majority of people (n = 133, 97.1%) had probable BPD and only two (1.5%) had CPTSD. Class 4 showed low probabilities of all symptoms.

### Analyses of differences among classes

#### Childhood abuse and invalidation environments

Table 4 presents the differences in childhood abuse and invalidating environment among latent classes. The comorbid class showed significantly higher PA, SA, EA, and PN scores. Class 2 was significantly higher than the low symptoms class in both SA and PN scores. Class 3 showed particularly high EN compared to the low symptom class, although there were no significant differences between it and Classes 1 and 2. Class 3 also showed higher scores in PA and EA compared to the Class 2. Class 1 showed the highest scores in the invalidating environment by both mother and father, followed by Classes 2 and 3, and Class 4.

**Table 4:** Mean score and differences in childhood abuse, invalidating environment, and attachment styles

		Class 1 (Comorbid) (*n* = 189)	Class 2 (Externalizing BPD) (*n* = 53)	Class 3 (Avoidant BPD) (*n* = 137)	Class 4 (Low Symptoms) (*n* = 120)	Chi-Square	Post-hoc
Childhood abuse	Physical abuse	8.00	3.32	4.34	2.74	257.17	1 > 3 > 2,4
	Emotional abuse	8.66	3.86	5.68	2.98	306.65	1 > 3 > 2,4
	Sexual abuse	6.87	1.67	1.60	0.57	331.83	1 > 2,3 > 4
	Physical neglect	7.83	4.78	5.19	3.30	244.58	1 > 2,3 > 4
	Emotional neglect	7.89	8.09	8.34	7.08	5.18	3 > 4
Invalidating environment	Maternal	26.23	18.31	19.69	15.42	198.61	1 > 2,3 > 4
	Paternal	27.36	18.28	19.38	15.37	206.94	1 > 2,3 > 4
Attachment style	Anxious	65.56	50.28	53.63	37.08	249.28	1 > 2,3 > 4
	Avoidant	58.62	54.13	61.30	53.03	15.89	1,3 > 4

*All post-hoc tests, except for emotion regulation, had *p*-values less than 0.001 (*df* = 3)

#### Attachment styles

Class 1 showed the highest scores in anxious attachment, followed by Classes 2 and 3, and Class 4. As for avoidant attachment, Class 3 showed the highest score, as it showed a similar level as Class 1.

#### Pathological personality traits

Table 5 lists the differences in pathological personality traits among classes. Class 1 showed significantly higher scores in all domains and facets. Meanwhile, Class 2 showed significantly higher antagonism domain scores than Classes 3 and 4. Additionally, Class 2 was higher in some facets than Class 3, such as callousness and grandiosity in the antagonism domain, risk taking in the disinhibition domain, and unusual beliefs and experiences in the psychoticism domain. By contrast, Class 3 showed higher scores than Class 2 in other facets, such as anxiousness in the negative affectivity domain as well as withdrawal and suspiciousness in the detachment domain.

**Table 5:** Mean score and differences in pathological personality traits

	Class 1 (Comorbid) (*n* = 189)	Class 2 (Externalizing BPD) (*n* = 53)	Class 3 (Avoidant BPD) (*n* = 137)	Class 4 (Low Symptoms) (*n* = 120)	Chi-Square	post-hoc
**Negative affectivity**	1.74	1.27	1.34	0.80	423.32	1 > 2,3 > 4
Emotional lability	1.78	1.34	1.49	0.94	223.91	1 > 2,3 > 4
Anxiousness	1.76	1.36	1.55	0.89	211.37	1 > 3 > 2 > 4
Depressivity	1.75	1.34	1.32	0.65	402.42	1 > 2,3 > 4
Hostility	1.74	1.24	1.36	0.88	197.86	1 > 2,3 > 4
Perseveration	1.77	1.14	1.22	0.84	316.54	1 > 2,3 > 4
Separation insecurity	1.61	1.20	1.08	0.61	248.09	1 > 2,3 > 4
Submissiveness	1.76	1.20	1.34	0.96	155.46	1 > 2,3 > 4
**Detachment**	1.74	1.30	1.42	1.03	248.01	1 > 2,3 > 4
Withdrawal	1.82	1.31	1.60	1.22	109.55	1 > 3 > 2,4
Intimacy avoidance	1.68	1.26	1.23	0.92	186.60	1 > 2,3 > 4
Anhedonia	1.72	1.37	1.44	0.93	226.71	1 > 2,3 > 4
Restricted affectivity	1.67	1.29	1.23	1.09	129.54	1 > 2,3 > 4
Suspiciousness	1.80	1.26	1.48	0.91	223.98	1 > 3 > 2 > 4
**Antagonism**	1.58	1.23	1.06	0.81	234.81	1 > 2 > 3 > 4
Deceitfulness	1.62	1.30	1.16	0.85	178.35	1 > 2,3 > 4
Manipulativeness	1.61	1.18	1.17	0.93	102.50	1 > 2,3 > 4
Callousness	1.58	1.20	0.96	0.70	273.12	1 > 2 > 3 > 4
Grandiosity	1.57	1.34	1.12	1.07	74.63	1 > 2 > 3,4
Attention seeking	1.54	1.15	0.97	0.69	156.01	1 > 2,3 > 4
**Disinhibition**	1.64	1.28	1.18	0.87	350.43	1 > 2,3 > 4
Rigid perfectionism	1.76	1.31	1.43	1.13	112.26	1 > 2,3,4 3 > 4
Distractibility	1.73	1.30	1.20	0.69	302.45	1 > 2,3 > 4
Impulsivity	1.66	1.15	1.06	0.76	253.33	1 > 2,3 > 4
Irresponsibility	1.62	1.28	1.16	0.78	232.71	1 > 2,3 > 4
Risk taking	1.51	1.30	1.05	0.89	151.54	1 > 2 > 3 > 4
**Psychoticism**	1.65	1.21	1.08	0.57	434.56	1 > 2,3 > 4
Unusual beliefs and experiences	1.55	1.21	0.98	0.51	306.14	1 > 2 > 3 > 4
Eccentricity	1.71	1.25	1.12	0.64	340.80	1 > 2,3 > 4
Cognitive and perceptual dysregulation	1.67	1.18	1.09	0.54	405.23	1 > 2,3 > 4

*The *p*-values of all post-hoc tests were less than 0.001 (*df* = 3)

## Discussion

This study identified CPTSD and BPD symptoms in an adult sample with childhood abuse experiences with the aim of comparing attachment styles and pathological personality traits that may differ in the development of each diagnosis in addition to variant aversive childhood experiences. Four classes were identified: Class 1 showed high probabilities for symptoms of PTSD, DSO, and BPD, which could be named as a “comorbid” class. Class 2 showed low probabilities for PTSD symptoms and a high probability for all BPD symptoms. It was characterized by externalizing personality traits. Therefore, we named it an “externalizing BPD” class. Class 3 showed moderate probabilities for PTSD symptoms and a high probability for loneliness as one of the BPD symptoms. Although it showed generally lower levels of BPD symptom severity than Class 2, it showed a prevalent avoidance-related attachment style and personality traits. Thus, it was named an “avoidant BPD” class. Lastly, Class 4 was named a “low symptom” class.

Our sample did not show a distinct CPTSD class but a comorbid class, which may be because childhood abuse is a common risk factor for PTSD, CPTSD, and BPD [9], and because high comorbidity of CPTSD and BPD has been reported for adults who experienced childhood abuse [3, 16]. These results were similar to those of previous studies, where the CPTSD and BPD comorbid class was shown to be dominant with varying symptom levels of BPD [14, 15].

Among the four groups, the comorbid class had the highest number of individuals, with a total of 189 (37.9%). This finding supports the high comorbidity of CPTSD and BPD [13–15] and the idea that childhood abuse is a common risk factor for both CPTSD and BPD [3, 9]. Overall, showing similar symptomatic proportions as Cloitre et al. [3], the majority of individuals were in the BPD classes when adding up the three symptomatic classes, thus leaving 120 individuals (24.0%) with low symptom probabilities. Some individuals who have experienced childhood abuse may not develop severe psychopathology due to various protective factors [58]; our study showed that these included low levels of adverse experiences, unstable attachment, and pathological personality traits.

The comorbid class had the highest probabilities for all symptoms along with higher scores for childhood abuse and invalidating environment, thus supporting the idea that the accumulation of these risk factors leads to the comorbidity of CPTSD and BPD [13, 16]. Both anxious and avoidant attachment styles were dominant in this class, confirming the findings of previous research [10, 23–27]. This class also had the highest scores on all domains and facets of pathological personality traits, thus indicating the severity of psychopathology [17]. We may therefore conclude that the most severe adulthood mental health consequences for childhood abuse are comorbid CPTSD and BPD, which require intensive psychosocial treatment [e.g., 59, 60].

In line with previous discussions showing that the presentation of BPD is highly heterogeneous [30, 61–63], our study identified subclasses of BPD. The two BPD classes showed moderate probabilities for DSO as well as a low probability for PTSD symptoms. The proportion of probable diagnoses for BPD was dominant, while those for CPTSD and PTSD were also relatively low, supporting that this group refers to subgroups of BPD. While the externalizing BPD class had a high probability of all BPD symptoms, the avoidant BPD showed loneliness as a dominant symptom while showing low probability for symptoms representing dissociation, shame or self-disgust, and suicidal or self-harm urges. Both classes also showed moderate DSO symptoms, suggesting that BPD symptoms and DSO symptoms might co-occur. This might be because that the ITQ could not fully differentiate DSO from the BPD symptoms as DSO symptoms show persistent negative self-concept and distant relational patterns while BPD symptoms show unstable fluctuations [3, 9]. This should be investigated by further studies using clinician rating assessments for CPTSD.

Both BPD classes showed similar levels of childhood sexual abuse and physical neglect and invalidating childhood environments, while the avoidant BPD class showed a substantially higher levels of emotional neglect and physical and emotional abuse than the externalizing BPD class. This is consistent with the findings of Müller et al. [64], which suggested that emotional neglect leads to social anxiety and avoidance. Emotional abuse or neglect was detected as a critical risk factor for people with BPD [5], which is consistent with the avoidant subgroup identified in our study, where this subgroup was also associated with avoidant attachment. In anxious attachment, both BPD classes showed high scores, which was also shown in previous studies, indicating that the ambivalent interpersonal relationship patterns in BPD are similar to the characteristics of anxious attachment [23].

The externalizing BPD class was associated with the pathological personality traits of the antagonism and disinhibition domains that make up the externalizing dimension [18], with significantly higher scores than the avoidant BPD class in callousness and grandiosity from the antagonism domain and in risk-taking from the disinhibition domain. This indicates that the externalizing BPD class can be understood as a group with a relatively deficient ability to empathize, an exaggerated sense of self, and a tendency to act according to their urges without regard for danger [8].

The avoidant BPD class scored higher than the externalizing BPD class in the pathological personality traits of anxiousness from the negative affectivity domain as well as those of withdrawal and suspiciousness from the detachment domain. The avoidant BPD class can be understood as a group of individuals with BPD who experience loneliness and anxiety related to negative perceptions of other people and environments who are detached and withdrawn from socio-interpersonal relationships [8]. Additionally, unusual beliefs and experiences from the psychoticism domain were significantly lower in the avoidant BPD class than they were in the externalizing BPD class, thus supporting that the avoidant BPD class may have lower levels of dissociative symptoms and uncontrollability.

People with externalizing BPD symptomatology may present brief psychotic episodes and/or abuse related dissociative symptoms. A previous study has reported that adult BPD patients show a high prevalence of hallucination, which is associated with PTSD symptoms, with authors emphasizing both PTSD and hallucination treatment in BPD [65]. Externalizing BPD might present more complicated treatment trajectories than avoidant BPD considering its potentially higher severities including suicidal and self-injurious behaviors. Additionally, dissociative and/or psychotic symptoms should be explored and reflected in the treatment plan that might represent activation of trauma memories unidentified, in line with a discussion that attachment trauma might present itself as implicit memories and that dissociation might affect symptom presentation [37].

Millon and colleagues [66] classified the following subtypes of BPD: discouraged BPD, impulsive BPD, petulant BPD, and self-destructive BPD. The results of the current study partially support this classification. While the externalizing subtype may represent impulsive, petulant, or self-destructive BPD, the avoidant subclass resembles the discouraged BPD subtype, which is characterized by passivity, separation anxiety, and a tendency to avoid others or social activities, ultimately causing one to experience loneliness and emptiness [66]. Prior empirical studies have also repeatedly identified subgroups of BPD: an “unstable” subtype and an “empty” subtype in a large undergraduate sample [67] as well as an “extravert/externalizing” and a “schizotypal/paranoid” subtype in a treatment study sample [63]. Gamache and colleagues [30] identified an externalizing (“impulsive”) and an internalizing (“depressivity”) subprofile. Our sample identified similar subclasses, namely, externalizing and avoidant, and these findings are consistent with the HiTOP model, as BPD is characterized by both externalizing and internalizing spectra [17, 18]. The heterogeneous expressions of BPD may vary depending on the spectrum of externalization-internalization and associated personality traits (antagonism vs. detachment); therefore, tailoring treatment according to the particular traits would have the largest treatment effects. For example, while stage-oriented treatment is needed in comorbid CPTSD/BPD and externalizing BPD starting from decreasing life-threatening behaviors prior to exposure-based interventions, avoidant BPD may benefit from targeting inhibited grief starting from childhood abuse-associated exposure treatment, for instance in stage 2 dialectical behavior therapy (DBT) [31]. Treatment of those in the CPTSD comorbid group should target PTSD symptoms in stage 2 DBT, and both BPD subgroups may benefit from targeting invalidating experiences or emotional abuse/neglect in stage 2 DBT, although PTSD symptoms are not present [e.g., 68].

The limitations of and suggestions for future research in this study are as follows: First, we were unable to compare the risk factors and pathological personality traits of CPTSD and BPD due to the absence of a distinct CPTSD class. Therefore, it is necessary to expand the discussion through variant sample studies including a clinical sample and using a structured clinical interview in the future. It should be also mentioned that the sample in this study primarily represented college educated early and midlife adults. A representative population study would enhance the generalizability of the findings. We also did not assess trauma in adulthood, nor did we assess the effect of polyvictimization in one’s lifetime. Moreover, as a cross-sectional study, the developmental course of CPTSD and BPD could not be confirmed. Further, using an online panel has the disadvantage of a high possibility of invalid participation [33]. However, although there were limitations, we applied a logical criterion when making judgments and excluded invalid responses.

## Conclusion

Childhood abuse and an invalidating environment were found to lead to devastating symptomatology, eventually yielding high comorbidity of CPTSD and BPD and externalizing-internalizing subgroups of BPD. In addition to assessing CPTSD and BPD symptoms, assessing attachment styles and pathological personality traits based on dimensional approaches may be the key to tailoring different treatment approaches for individuals and to anticipate treatment trajectories.

## Data Availability

The datasets generated and/or analysed during the current study are not publicly available because the participants of this study did not agree for their data to be shared publicly but are available from the corresponding author on reasonable request.
